# Modulation of gut microbiota and intestinal barrier by lotus seed, jujube, and longan aril in senna leaf-induced diarrhea in mice

**DOI:** 10.3389/fmicb.2026.1796355

**Published:** 2026-07-07

**Authors:** Fangkun Zhao, Rongrong Xiao, Xiao Li, Qilei Xin, Xiangmei Chen, Dan Zhou, Wencui Liu, Jiahui Zhang, Wei Lu, Chunping Jiang, Guang Zhang

**Affiliations:** 1 Division of Hepatobiliary and Transplantation Surgery, Department of General Surgery, Nanjing Drum Tower Hospital, Clinical College of Nanjing University of Chinese Medicine, Nanjing, China; 2 Jinan Microecological Biomedicine Shandong Laboratory, Building 1, Jinan Medical and Health Science and Technology Innovation Industrial Park, Jinan, China; 3 State Key Laboratory of Pharmaceutical Biotechnology, Division of Hepatobiliary and Transplantation Surgery, Department of General Surgery Nanjing Drum Tower Hospital, Affiliated Hospital of Medical School, Nanjing University, Nanjing, China; 4 Department of Hepatobiliary and Pancreatic Surgery, The Second Affiliated Hospital of Fujian Medical University, Quanzhou, Fujian, China; 5 Jiangsu Province Hospital of Chinese Medicine, Affiliated Hospital of Nanjing University of Chinese Medicine, Nanjing, Jiangsu, China; 6 Renhuai People's Hospital, Renhuai, Guizhou, China

**Keywords:** diarrhea, gut microbiota, intestinal mucosal barrier, medicinal and edible homology, senna leaf model

## Abstract

**Objective:**

This study aimed to investigate the regulatory effects of lotus seed, jujube, and longan aril on the gut microbiota structure and barrier function in a senna leaf-induced diarrhea mouse model.

**Methods:**

Diarrhea was induced in ICR mice using senna leaf extract. Mice received aqueous extracts of lotus seed, jujube, or longan aril for treatment. Intestinal motility was assessed through fecal consistency scoring and the charcoal propulsion test. Colon tissues were collected for histopathological examination using H&E staining, as well as immunohistochemical and Western blot analyses of aquaporin 3 (AQP3) and sodium-hydrogen exchanger 8 (NHE8). Gut microbiota composition was analyzed via 16S rRNA sequencing.

**Results:**

All three herbal interventions significantly reduced the loose stool rate and diarrhea index (*p* < 0.05), inhibiting small intestinal hypermotility. Histological analysis revealed an increase in goblet cell numbers and improved crypt architecture in the colon. Immunohistochemical evaluation indicated downregulation of AQP3 and upregulation of NHE8 expression, which was confirmed by Western blot analysis. 16S rRNA sequencing demonstrated that all treatments restored *α*-diversity (Shannon index, *p* < 0.05). *β*-diversity analysis revealed that longan aril induced a more extensive reshaping of gut microbial community structure compared to lotus seed and jujube, which exhibited a “phased” restoration. The relative abundance of potential pathogens (e.g., *Akkermansia muciniphila*, *Citrobacter* spp.) significantly decreased, while beneficial short-chain fatty acid-producing bacteria (e.g., *Blautia coccoides*, *Faecalibaculum rodentium*, *Alloprevotella rava*) were enriched. Unique protective taxa, such as nitrogen-fixing *Azospirillum* and antioxidant *Deinococcus*, emerged in specific treatment groups.

**Conclusion:**

Lotus seed, jujube, and longan aril synergistically alleviated diarrhea via multiple pathways, including modulation of gut microbiota structure, repair of barrier function, balance of water-electrolyte metabolism, and inhibition of intestinal hypermotility. This study provides a scientific foundation for the clinical application of medicinal and edible homology substances, as well as the development of microecological agents.

## 1 Introduction

Diarrhea is a prevalent global health issue, significantly impacting morbidity and mortality, particularly among children and the elderly. According to estimates by the World Health Organization, diarrhea remains a leading cause of death worldwide, responsible for approximately 1.5 million annual fatalities, nearly 500,000 of which occur in children under the age of five. The condition can be classified into acute and chronic forms based on duration and etiology. Acute diarrhea is often caused by bacterial, viral, or parasitic infections, whereas chronic diarrhea frequently arises from non-infectious conditions such as irritable bowel syndrome, inflammatory bowel disease, or functional gastrointestinal disorders (Neurath, 2017). While treatments like oral rehydration therapy, antibiotics, and probiotics are widely utilized, they often present limitations including antibiotic resistance, microbiota disruption, and variable efficacy (Ríos-Covián et al., 2016). Consequently, there is an urgent need for safe, effective, and sustainable therapeutic alternatives.

In recent years, the role of gut microbiota in diarrheal pathogenesis has gained increasing attention. Gut dysbiosis—characterized by reduced diversity and altered composition of microbial communities—has been closely associated with impaired intestinal barrier function, dysregulated immune responses, and abnormal water-electrolyte transport. Key molecular players in these processes include aquaporin 3 (AQP3), a water channel protein involved in luminal water secretion, and sodium-hydrogen exchanger 8 (NHE8), which mediates sodium absorption and intracellular pH regulation. In diarrheal states, AQP3 is frequently upregulated, promoting excessive water efflux, while NHE8 expression may be altered, contributing to electrolyte imbalances. Thus, restoring gut microbiota homeostasis and repairing the intestinal barrier represent promising therapeutic strategies for diarrhea management.

Traditional Chinese Medicine offers a unique perspective with the concept of “medicinal and edible homology,” referring to natural substances that serve dual roles as food and medicine. This approach emphasizes the use of dietary components for disease prevention and treatment, aligning with holistic and preventive healthcare principles. Lotus seed (*Nelumbo nucifera*), jujube (*Ziziphus jujuba*), and longan aril (*Dimocarpus longan*) are three representative substances traditionally employed to strengthen the spleen, replenish qi, and alleviate diarrhea associated with spleen-stomach weakness. Modern pharmacological studies have identified bioactive components in these materials, such as polysaccharides, polyphenols, and cyclic nucleotides, demonstrating anti-inflammatory, antioxidant, and immunomodulatory properties. However, their effects on gut microbiota structure and intestinal barrier function during diarrhea remain insufficiently understood, with underlying mechanisms not yet systematically elucidated (Table 1).

**Table 1:** qPCR primer sequences.

Gene name	Primer sequence (5′ → 3′)
NHE2	F: TTACCTAAGAACACAAAGCTTCCAG
	R: GAGCACAGTGGTTCCAACATC
NHE3	F: CTTCGCCTTCCTGCTGTCCTTG
	R: GATGGTGATGCCGAAGTTGGTG
NHE8	F: TCTCGGAAGCTCGCAGGATA
	R: CAGACCCCTGTGGGCTTATT
AQP3	F: GAGATGCTTCACATCCGCTACCG
	R: CCAGCCACCAAGATGCCAAGG
AQP4	F: GGAGCTACATGGAGGTGGAGGAC
	R: GATGCGGTTCAGCTCCACGATG
β-actin	F: GTGCTATGTTGCTCTAGACTTCG
	R: ATGCCACAGGATTCCATACC

To address this knowledge gap, a senna leaf-induced diarrhea mouse model was employed to evaluate the therapeutic effects of lotus seed, jujube, and longan aril. Diarrheal phenotypes, intestinal motility, histopathological changes, and the expression of AQP3 and NHE8 in colonic tissues were assessed. Additionally, 16S rRNA sequencing was utilized to analyze gut microbiota composition and diversity. This study aims to provide a comprehensive understanding of how these three natural substances alleviate diarrhea through microbiota modulation and barrier repair, thereby offering scientific support for their clinical application and the development of microbiota-targeted interventions.

## 2 Materials and methods

### 2.1 Animals and ethics

Sixty-three female ICR mice (6 weeks old, weighing 18–22 g) were acquired from the Comparative Medicine Center of Yangzhou University (production license no. SCXK [Su] 2022–0009). Mice were housed individually in isolation cages with unrestricted access to food and water. The laboratory maintained a temperature of (24 ± 1) °C and relative humidity of 40–80%. All procedures received approval from the Animal Ethics and Welfare Committee of NJU (SYXK2019-0056) and adhered to institutional guidelines. Experiments commenced after a 3-day acclimatization period.

### 2.2 Preparation of herbal extracts

Lotus seed, jujube, and longan aril were sourced from Bozhou Jingwan Chinese Herbal Pieces Factory. Each herb was decocted twice in ultrapure water (1,000 mL each time for 40 min). The combined filtrates were concentrated to 50 mL, resulting in a final concentration of 1.0 g crude drug/mL. Senna leaf decoction was prepared similarly for diarrhea induction. All extracts were stored at 4 °C (Table 2).

**Table 2:** Detailed list of up- and down-regulated gut bacteria after lotus seed treatment (species level).

Species	Change	Effects/Comments
*Akkermansia muciniphila*	Down	/
*Muribaculum intestinale*	Down	Belongs to Bacteroidetes phylum, strictly anaerobic Gram-negative. Found to have significant immunomodulatory functions, particularly its metabolite MiCL-1 can induce pro-inflammatory cytokines like TNF-α, IL-6, and IL-23, but no significant effect on anti-inflammatory IL-10 (Bang et al., 2023).
*Ruminococcus callidus*	Down	/
*Anaerotruncus colihominis*	Up	Regulates intestinal epithelial barrier function by producing SCFAs like butyrate and acetate, and may affect CNS autoimmune responses (Bianchimano et al., 2022).
*Blautia coccoides*	Up	Promotes colonic mucosal growth and function by producing SCFAs like propionate and acetate. Increases the proportion of Gram-positive bacteria in the gut (Yang et al., 2022, Holmberg et al., 2024, Niu et al., 2024, Wang B. et al., 2024).
*Hungatella hathewayi*	Up	/
*Lachnoclostridium edouardi*	Up	Involved in SCFA production, maintenance of gut health, potential disease marker.(Song et al., 2021)
*Marvinbryantia formatexigens*	Up	Modulates gut microbiota composition, promotes growth of beneficial bacteria, inhibits harmful bacteria, thus improving gut health. Can produce beneficial metabolites like SCFAs, e.g., butyrate.(Chen et al., 2024, Han et al., 2022, Lu et al., 2024)

### 2.3 Experimental design and treatment

After 3 days of acclimation, mice were randomly assigned to seven groups (*n* = 9 per group): (1) Normal group (pure water); (2) Model group (senna leaf decoction, 10 g/kg, following pure water gavage); (3) Montmorillonite powder group (positive control, 1 g/kg); (4) Berberine hydrochloride group (positive control, 0.1 g/kg); (5) Model + Lotus Seed group; (6) Model + Jujube group; and (7) Model + Longan Aril group (groups 5–7 received 10 g/kg of respective extracts). Mice were fasted for 6 h (water accessible) and then administered the corresponding treatments once daily for 14 days. Senna leaf decoction (10 g/kg) was administered 0.5 h after each treatment. Fecal characteristics and overall health were monitored throughout the intervention.

The dosage of 10 g/kg for lotus seed, jujube, longan aril, and berberine hydrochloride was selected based on pre-experimental findings and previous literature. In pre-experiments, three doses (5, 10, and 20 g/kg) of each herbal extract were tested, revealing that 10 g/kg provided the optimal anti-diarrheal effect without any evident toxic side effects. Conversely, 5 g/kg showed limited efficacy, and 20 g/kg did not enhance the therapeutic outcome (Supplementary Figures S3, S4). This dosage aligns with the commonly employed range for medicinal and edible homology substances in mouse diarrhea models (Madjirebaye et al., 2024; Lu et al., 2024). The montmorillonite powder dose (1 g/kg) was determined based on clinical dose conversion equivalents between mice and humans. Preliminary validation of gut microbiota dysbiosis in the model is shown in Supplementary Figure S2.

### 2.4 Assessment of diarrhea phenotypes

To quantify diarrhea severity and intestinal motility, fecal consistency was scored daily using a standardized 4-point scale (0 = normal, 1 = soft, 2 = loose, 3 = watery diarrhea). The diarrhea index for each group was calculated based on the consistency scores and fecal frequency throughout the intervention. Concurrently, small intestinal propulsion was assessed using the charcoal propulsion test. Mice received an oral administration of 0.2 mL of charcoal ink suspension 0.5 h following the senna leaf challenge. After 25 min, the entire small intestine was carefully excised and straightened. The propulsion rate was calculated as the ratio of the distance traveled by the charcoal front to the total length of the small intestine, expressed as a percentage (propulsion rate = [charcoal distance / total intestinal length] × 100%) (Table 3).

**Table 3:** Gut bacteria that disappeared from the unique flora of the model group after lotus seed treatment.

Species	Effects/Comments
*Anaerofilum*	Harmful bacteria associated with gastrointestinal inflammation (Madjirebaye et al., 2024).
*Candidatus_Obscuribacter*	In a PM2.5 exposure-induced rat thyroid dysfunction model, showed significant correlation with urine metabolites. Positively correlated with taurocholic acid levels, suggesting a role in bile acid metabolism related to thyroid dysfunction (Dong X. et al., 2022).
*Carboxydocella*	/
*Citrobacter*	Opportunistic pathogen capable of causing various infections (UTI, meningitis, respiratory, GI infections, neonatal sepsis), especially in immunocompromised patients (Jolivet et al., 2024).
*Cohnella*	/
*Desulfonatronum*	/
*Haloferula*	/
*Hydrogenibacillus*	/
*Marinilabilia*	/
*Moorella*	/
*Persicirhabdus*	Possible producer of tetrodotoxin (TTX), a potent neurotoxin causing human poisoning (Biessy et al., 2024).
*Prolixibacter*	/
*Prosthecobacter*	16S rRNA sequencing suggests potential tetracycline-resistant bacteria (Huang et al., 2014).
*Rodentibacter*	*Rodentibacter pneumotropicus* is an invasive Gram-negative mouse pathogen causing severe infections in various animals (Fertey et al., 2020).
*Roseibacillus*	/
*Tepidibacillus*	/
*Terrimicrobium*	/
*Ureibacillus*	Its loss positively correlated with reduction in tetracycline resistance genes (Liu et al., 2020).
*Virgibacillus*	/

### 2.5 H&E staining

Colon tissues were fixed in 4% paraformaldehyde, embedded in paraffin, sectioned, and stained with hematoxylin and eosin (H&E). For immunohistochemistry (IHC), sections underwent antigen retrieval in citrate buffer (pH 6.0), were blocked with goat serum, and incubated overnight at 4 °C with primary antibodies against AQP3 (1:200) and NHE8 (1:200). Visualization was achieved using HRP-conjugated secondary antibodies and DAB substrate. Positive cells were quantified using a light microscope.

### 2.6 qPCR

Quantitative PCR (qPCR) was conducted with ChamQ Blue Universal SYBR Master Mix. A 20 μL reaction system was prepared (10 μL 2 × Master Mix, 0.4 μL each of 10 μM forward and reverse primers, and ≤2 μL of template cDNA). Amplification followed a standard program (pre-denaturation at 95 °C for 30 s; 40 cycles: 95 °C for 5 s and 60 °C for 30 s; with melt curve analysis). RNase-free consumables were utilized throughout the experiment. No-template controls (NTC) and No RT controls were prepared to exclude contamination. Primer final concentration was optimized to 0.1–1.0 μM, and amplification efficiency was confirmed by a single peak in the melt curve (indicating specificity), as well as through the ΔΔCt method. All primers were synthesized by Tsingke Biotechnology Co., Ltd. (Beijing, China). Primer specificity was confirmed by single peak in melt curve analysis, and amplification efficiency was verified to be between 90 and 110% via standard curve method (Table 4).

**Table 4:** Newly appeared gut bacteria after lotus seed treatment compared to the union of control and model groups.

Species	Effects/Comments
*Azospira*	Bacteria capable of using nitrate and nitrite as electron acceptors, can fix nitrogen, converting N_2_ to plant-usable forms, promoting plant growth, disease resistance, enhancing systemic resistance in tobacco to bacterial wilt (Wang et al., 2022).
*Azospirillum*	Fixes atmospheric N_2_ into NH₃, increasing plant nitrogen content; synthesizes various plant hormones including abscisic acid, significantly affecting root development, improving water/nutrient uptake (Shang et al., 2024, Wang S. et al., 2024).
*Comamonas*	/
*Desulfonatronospira*	/
*Ideonella*	/
*Lachnoanaerobaculum*	This species may protect the host from pathogens and chronic inflammation. L-carnitine supplementation can increase its levels, exerting cardioprotective effects (Volodina et al., 2022).
*Libanicoccus*	/
*Massilia*	/
*Microbacterium*	This strain has high fiber utilization capacity, aiding Diqing Tibetan pigs in efficient dietary fiber utilization (Yang L. et al., 2025).
*Paenisporosarcina*	Genomic content of this bacterium significantly reduced in rheumatoid arthritis patients (Ormseth et al., 2020).
*Pseudomonas*	*Pseudomonas koreensis* promotes rice growth and reduces cadmium (Cd) content; Cd is easily absorbed by rice entering the food chain, posing human health risks (Wu et al., 2025).
*Riemerella*	Relative abundance of this strain significantly decreased in genetically obese mice (Dong S. et al., 2022).
*Shinella*	/
*Slackia*	/
*Vulcaniibacterium*	/

### 2.7 Western blot analysis

For protein analysis, mouse colon tissue was homogenized by grinding with lysate on ice. The supernatant was collected after centrifugation, and protein concentration was quantified. A sample of 10 μg protein was denatured by boiling and subjected to SDS-PAGE electrophoresis. The membrane was immediately transferred using the wet transfer method and blocked with 5% skim milk powder for 2 h. Following TBS-T washes, primary antibodies (AQP3 1:1000, NHE8 1:1000) and an internal reference (*β*-actin 1:20000) were added according to the respective molecular weights and incubated overnight at 4 °C. The following day, after further TBS-T washes, an HRP-labeled goat anti-rabbit IgG secondary antibody was added and incubated for 1 h. Protein bands were visualized with enhanced chemiluminescence (ECL) reagent (EMD Millipore, Billerica, MA, USA) (Table 5).

**Table 5:** Detailed list of up- and down-regulated gut bacteria after jujube treatment (species level).

Species	Change	Effects/Comments
*Akkermansia muciniphila*	Down	/
*Ruminococcus callidus*	Down	/
*Dorea longicatena*	Up	Proportion of *Dorea longicatena* increased in patients in remission after ileocolonic resection (ICR) for Crohn’s disease (CD); ICR greatly impacts the gut microbial ecosystem (Mondot et al., 2016).
*Faecalibaculum rodentium*	Up	F. rodentium modulates retinoic acid signaling and eosinophils, inhibits IFN-*γ* production, affects intestinal epithelial cell proliferation and MHCII expression; its metabolites, especially butyrate, inhibit tumor cell proliferation by suppressing calcineurin and NFATc3 activation, showing potential anti-tumor effects (Guo et al., 2022, Zagato et al., 2020).
*Robinsoniella peoriensis*	Up	/
*Staphylococcus lentus*	Up	/

### 2.8 16S rRNA analysis

Fecal samples were collected on day 14, and total DNA was extracted using the HiPure Soil DNA Mini Kit. The V3–V4 region of the 16S rRNA gene was amplified with primers 338F/806R and sequenced on an Oxford Nanopore PromethION platform (SQK-LSK114 kit). Raw reads were quality-filtered, clustered into amplicon sequence variants (ASVs), and analyzed for *α*-diversity (Shannon index), *β*-diversity (PCA), and taxonomic composition using the Bena Cloud Platform (Wuhan Bena Technology Co., Ltd.).

### 2.9 Statistical methods

All quantitative data are presented as mean ± standard deviation (mean ± SD). Statistical analyses were performed using SPSS 26.0 software (IBM, USA). Differences between two groups were evaluated using Student’s t-test, while comparisons among multiple groups were conducted using one-way analysis of variance (ANOVA). A *p*-value of < 0.05 was considered statistically significant (Table 6).

**Table 6:** Gut bacteria that disappeared from the unique flora of the model group after jujube treatment.

Species	Effects/Comments
*Anaerofilum*	Harmful bacteria associated with gastrointestinal inflammation (Madjirebaye et al., 2024).
*Candidatus_Xiphinematobacter*	/
*Carboxydocella*	/
*Cloacibacterium*	/
*Cohnella*	/
*Desulfocurvus*	/
*Hydrogenibacillus*	/
*Marinilabilia*	/
*Persicirhabdus*	Possible producer of tetrodotoxin (TTX), a potent neurotoxin causing human poisoning (Biessy et al., 2024).
*Prosthecobacter*	16S rRNA sequencing suggests potential tetracycline-resistant bacteria (Huang et al., 2014).
*Rodentibacter*	Rodentibacter pneumotropicus is an invasive Gram-negative mouse pathogen causing severe infections (Fertey et al., 2020).
*Roseibacillus*	/
*Tepidibacillus*	/
*Terrimicrobium*	/
*Virgibacillus*	/

## 3 Results

### 3.1 Improvement of diarrhea phenotype

#### 3.1.1 Changes in mouse physical condition and pathological features

**Figure 1:**
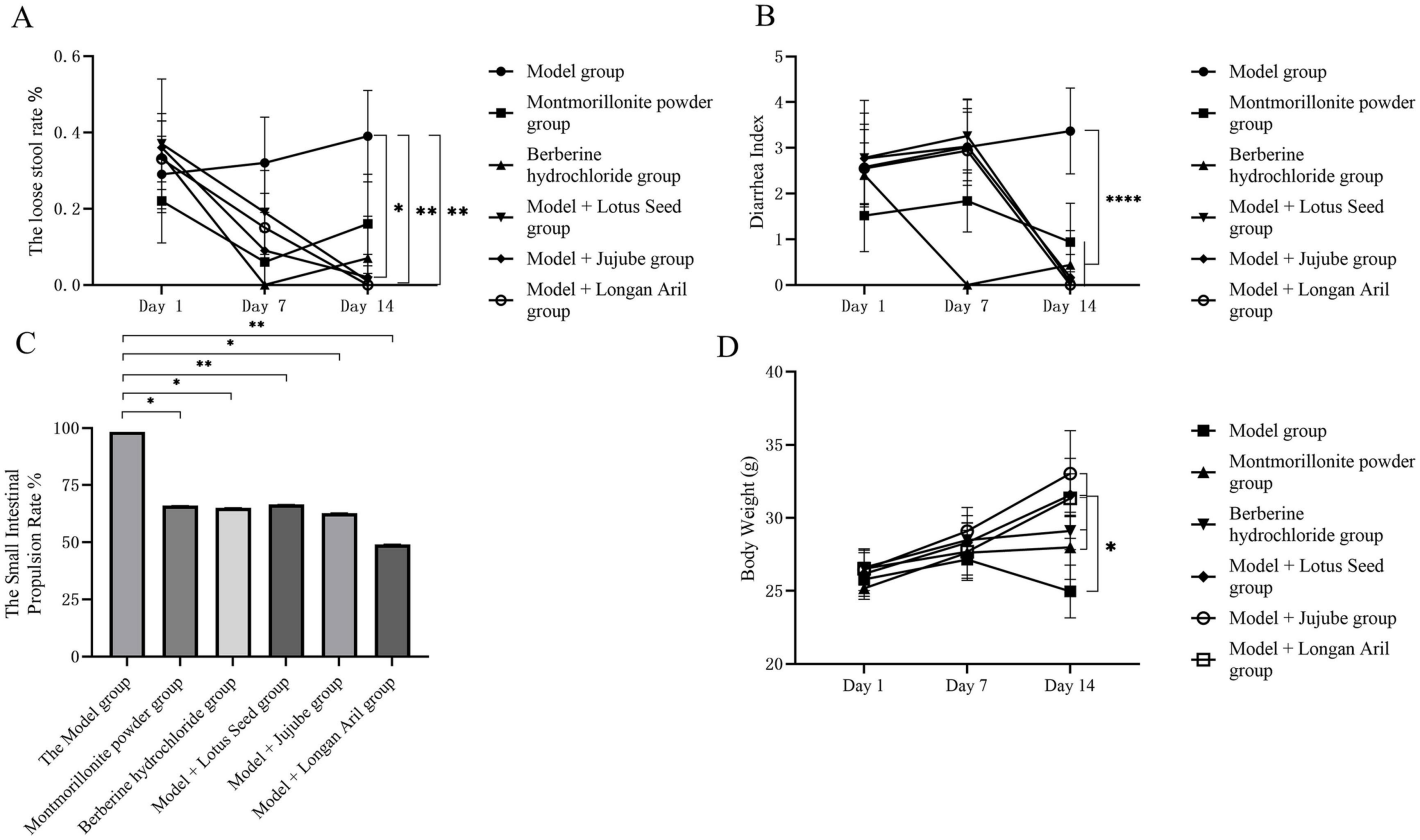
Effects of lotus seeds, jujubes, and longan aril on senna leaf-induced diarrhea in mice. **(A)** Loose stool rate, **(B)** Diarrhea index, **(C)** Small intestinal propulsion rate, **(D)** Body weight changes in mice during the 14-day intervention period. Compared to the model group, *p < 0.05, **p < 0.01, ****p < 0.0001.

Mice in the Normal group exhibited normal feeding behavior, flexible movement, glossy fur, and consistently formed stools throughout the experiment. In contrast, mice in the Model group began displaying signs of loose stools from day 3, accompanied by weight loss, emaciation, dull fur, sluggish movement, huddling, abdominal retraction, and licking of the abdomen. Mice in the drug treatment groups showed slight improvements in demeanor and some weight gain by day 7 of administration. Subsequently, the aforementioned symptoms and signs gradually alleviated. Mice in the positive control group, as well as those receiving lotus seed, jujube, or longan aril, largely returned to a normal state, characterized by improved feeding, restored fur condition, and disappearance of abdominal licking. Changes in body weight across the various groups are depicted in Figure 1D.

#### 3.1.2 Small intestinal propulsion function

Results displayed in Figure 1C indicate that both charcoal propulsion distance and rate were significantly reduced in the drug treatment groups, demonstrating statistical significance (*p* < 0.05); however, no significant differences were found among the drug groups themselves (*p* > 0.05).

Lotus seed, jujube, and longan aril significantly improved fecal characteristics, evidenced by a reduced loose stool rate in senna leaf-induced diarrheal mice, and markedly inhibited the small intestinal propulsion rate (*p* < 0.05), confirming a clear anti-diarrheal effect (Table 7).

**Table 7:** Newly appeared gut bacteria after jujube treatment compared to the union of control and model groups.

Species	Species
*Anaerosporobacter*	Abundance of this species is negatively correlated with the occurrence of pediatric multiple sclerosis (Tremlett et al., 2021).
*Globicatella*	/
*Johnsonella*	Studies on gut microbiota and atherosclerosis show that the content of this species is negatively correlated with carotid plaque (Wang et al., 2023).
*Lachnoanaerobaculum*	This species may protect the host from pathogens and chronic inflammation. L-carnitine supplementation can increase its levels, exerting cardioprotective effects (Volodina et al., 2022).
*Paeniclostridium*	/
*Paenisporosarcina*	Genomic content of this bacterium significantly reduced in rheumatoid arthritis patients (Ormseth et al., 2020).
*Paraclostridium*	Germ-free mice colonized with the amino acid-fermenting bacterium Paraclostridium bifermentans survived *C. difficile* infection with reduced disease severity (Girinathan et al., 2021).
*Petrimonas*	/
*Proteus*	/

#### 3.1.3 Changes in fecal characteristics

The loose stool rate and diarrhea index were compared between groups, with results shown in Figures 1A,B. On day 1 of treatment, no statistically significant differences in the loose stool rate were found between any intervention group and the diarrhea model group (*p* > 0.05). The diarrhea index was significantly reduced only in the Montmorillonite powder group (*p* < 0.05), while no differences were observed in the other groups (*p* > 0.05). By day 14 of treatment, the loose stool rate in the medicinal and edible homology substance groups (Lotus Seed, Jujube, Longan Aril) was significantly lower than that in the Model group (*p* < 0.01), with the diarrhea index showing a highly significant decrease across all treatment groups (*p* < 0.0001).

### 3.2 Pathological changes in the intestinal mucosa

#### 3.2.1 H&E staining shows repair of intestinal villus damage

**Figure 2:**
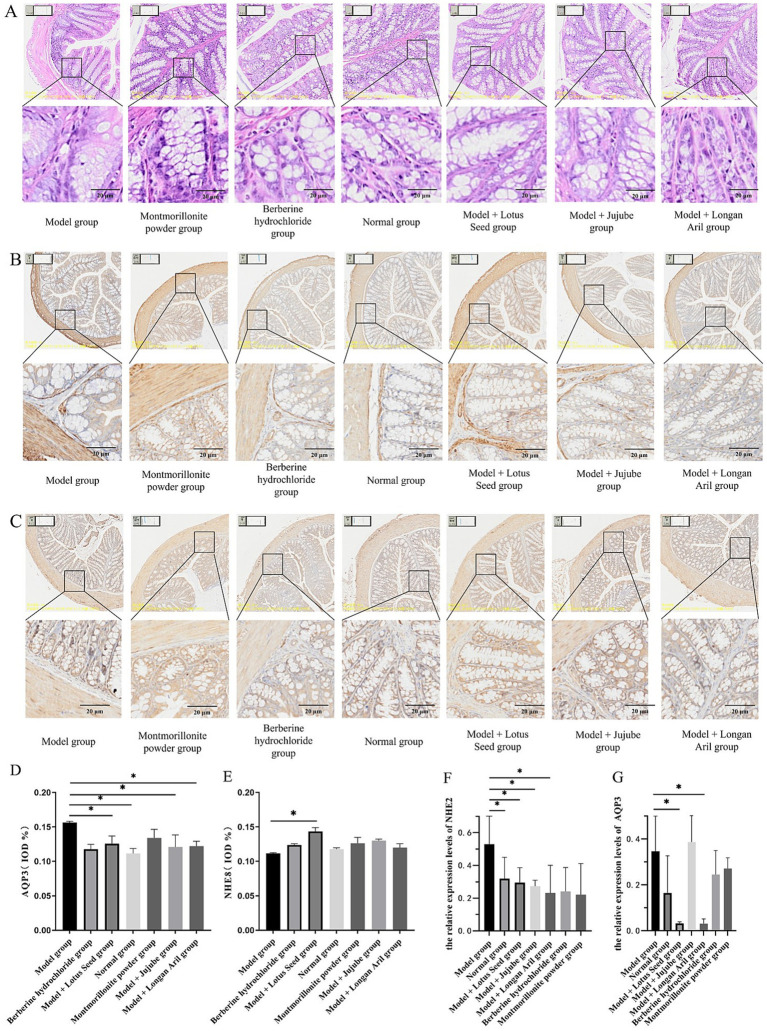
Effects of lotus seeds, jujubes, and longan aril on intestinal histopathological morphology, inflammation-related proteins, and gene expression in mice with intestinal inflammation. **(A)** H&E images, **(B)** IHC images of AQP3, **(C)** IHC images of NHE8, **(D)** IOD of AQP3, **(E)** IOD of NHE8, **(F)** Relative NHE2 mRNA expression, **(G)** Relative AQP3 mRNA expression. Compared to the model group, *p < 0.05.

As illustrated in Figure 2A, compared to the Model group, pathological damage in the colon tissue of mice in the lotus seed, jujube, and longan aril intervention groups was significantly improved. The epithelial cells in the mucosal layer were neatly arranged with an intact structure, the number of goblet cells increased, and crypt morphology was largely restored. These results confirm that the three herbs effectively repaired colonic mucosal damage in senna leaf-induced diarrheal mice (*p* < 0.05) (Table 8).

**Table 8:** Detailed list of up- and down-regulated gut bacteria after longan aril treatment (species level).

Species	Change	Effects/Comments
*Alloprevotella_rava*	Up	Produces SCFAs; strong negative correlation with pathological symptoms in experimental autoimmune encephalomyelitis (EAE); oral *A. rava* has beneficial effects on EAE; increased abundance raises butyrate/valerate levels, helping prevent colorectal tumors (Han et al., 2024, Xue et al., 2020).
*Staphylococcus_lentus*	Up	/
*Staphylococcus_nepalensis*	Up	/
*Akkermansia muciniphila*	Down	/
*Eisenbergiella tayi*	Down	/
*Ruminococcus flavefaciens*	Down	/

#### 3.2.2 Immunohistochemistry: upregulation of tight junction protein expression

Results presented in Figures 2B,D indicate that, compared to the Model group, the number of AQP3-positive cells and their spatial distribution density in the colon of mice treated with lotus seed, jujube, and longan aril were significantly reduced (*p* < 0.05). Notably, AQP3 expression in the Lotus Seed group did not differ statistically from that in the Normal group (*p* > 0.05). AQP3, a key channel protein involved in intestinal water absorption, is abnormally upregulated in the senna leaf-induced diarrhea model, directly promoting excessive water secretion into the intestinal lumen. The medicinal and edible homology substances normalized intestinal water-electrolyte absorption homeostasis by downregulating AQP3 expression to physiological levels, thereby alleviating diarrhea. IHC results confirmed that AQP3 protein expression in the colon was significantly downregulated in all three treatment groups compared to the Model group (*p* < 0.05). In contrast, qPCR results indicated that AQP3 mRNA expression was decreased in the lotus seed and longan aril groups (*p* < 0.05) but increased in the jujube group (*p* < 0.05). Western blot analysis further corroborated that AQP3 protein expression was lower in all treatment groups than in the Model group, although it remained elevated compared to the Normal group, except in the lotus seed group (Table 9).

**Table 9:** Gut bacteria that disappeared from the unique flora of the model group after longan aril treatment.

Species	Effects/Comments
*Anaerofilum*	Harmful bacteria associated with gastrointestinal inflammation (Madjirebaye et al., 2024).
*Candidatus_Xiphinematobacter*	/
*Carboxydocella*	/
*Catonella*	Relative abundance of this species increased in astronauts during flight, potentially leading to virus reactivation, posing health threats (Urbaniak et al., 2020).
*Cloacibacterium*	/
*Cohnella*	/
*Desulfocurvus*	/
*Geobacter*	/
*Hydrogenibacillus*	/
*Listeria*	*Listeria monocytogenes* widely used as a model for studying pathogenesis and host defense against Gram-positive intracellular bacteria (Yang B. et al., 2025).
*Marinilabilia*	/
*Moorella*	/
*Persicirhabdus*	Possible producer of tetrodotoxin (TTX), a potent neurotoxin causing human poisoning (Biessy et al., 2024).
*Prosthecobacter*	16S rRNA sequencing suggests potential tetracycline-resistant bacteria (Huang et al., 2014).
*Roseibacillus*	/
*Tepidibacillus*	/
*Terrimicrobium*	/
*Ureibacillus*	Its loss positively correlated with reduction in tetracycline resistance genes (Liu et al., 2020).

Results shown in Figures 2C,E demonstrate that the number and distribution density of NHE8-positive cells in the colon of mice in the Lotus Seed intervention group were significantly increased compared to the Model group (*p* < 0.05), with expression levels comparable to those in the Normal group (*p* > 0.05). NHE8, a member of the SLC9A family, is located on the apical membrane of intestinal epithelial cells and mediates sodium absorption and intracellular pH regulation through H⁺-Na⁺ exchange. The upregulation of NHE8 under diarrheal conditions may represent a compensatory response to impaired sodium absorption, enhancing transmembrane sodium transport to counteract electrolyte loss resulting from CFTR channel-mediated Cl^−^ secretion.

### 3.3 Gene expression of inflammatory pathway

As illustrated in Figures 2F,G, the relative expression levels of NHE2 and AQP3 in the Model + Lotus Seed and Model + Longan Aril groups were significantly decreased compared to the Model group (*p* < 0.05). Interestingly, the Model + Jujube group exhibited a significant decrease in NHE2 expression, alongside a significant increase in AQP3 expression (*p* < 0.05).

The profiles of active components in red jujube fundamentally differ from those in lotus seed and longan aril, which contributes to their distinct regulatory effects on the targets. Lotus seeds and longan aril likely possess shared active constituents, such as specific flavonoids and polysaccharides, capable of concurrently suppressing the transcription or expression of both NHE2 and AQP3 through the same signaling pathways (e.g., NF-κB and MAPK), aligning with the anticipated regulatory direction. Conversely, red jujube appears to contain unique active components—such as jujube polysaccharides, cyclic adenosine monophosphate (cAMP), or specific alkaloids—that selectively modulate the targets. These components may inhibit NHE2 expression, consistent with the effects noted for the other two materials, while simultaneously activating upstream regulatory pathways of AQP3 (e.g., PI3K/Akt or AMPK signaling) or alleviating transcriptional repression, resulting in AQP3 upregulation. This leads to a divergent regulatory outcome that contrasts with expected effects.

### 3.4 Western blot analysis results of lotus seed on gut microbiota in senna leaf-induced diarrhea model mice

**Figure 3:**
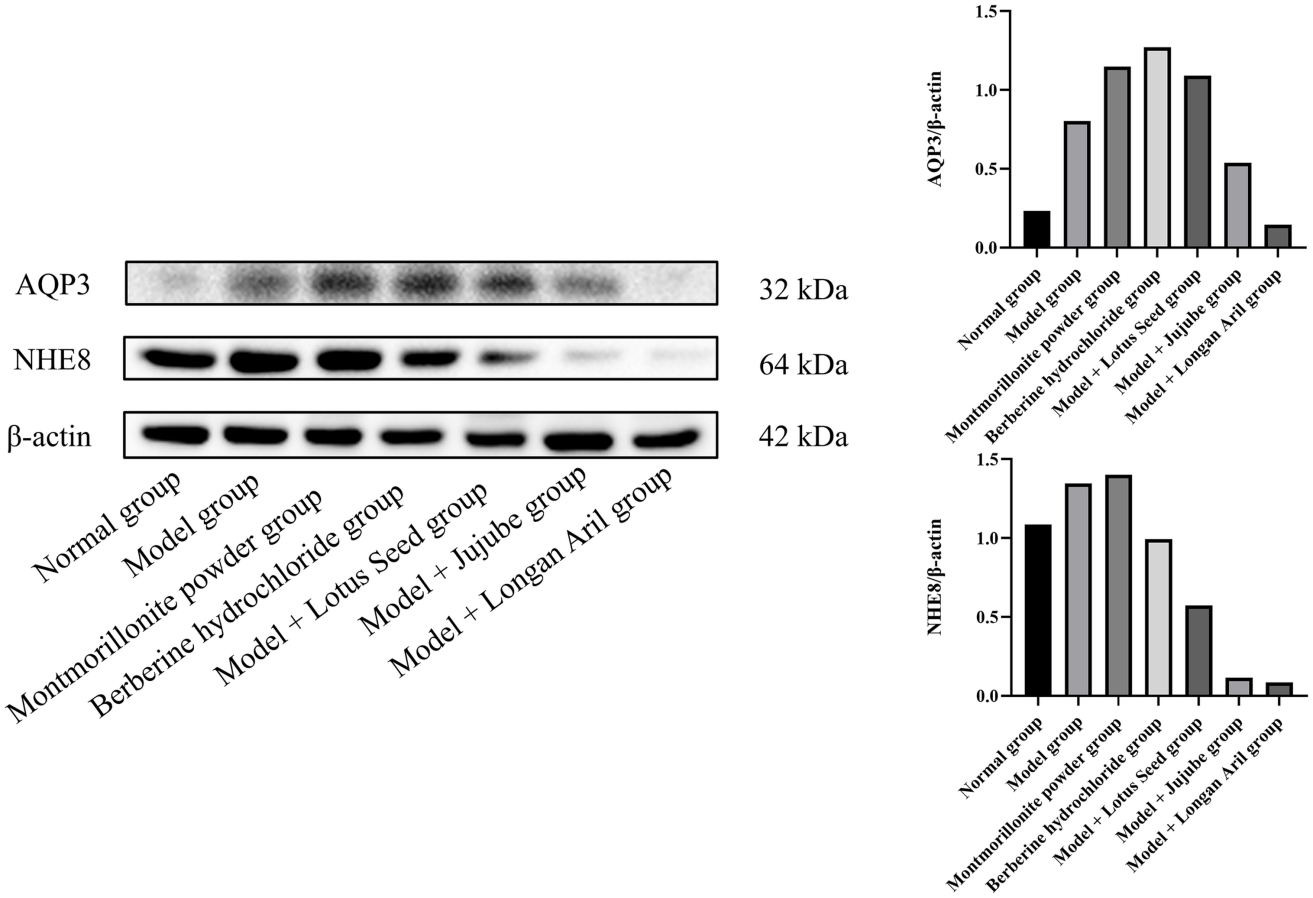
Effects of lotus seeds, jujubes, and longan aril on the levels of AQP3 and NHE8 in the colons of mice with intestinal inflammation. The protein expression levels of AQP3 and NHE8 were detected by Western blot analysis, and the grayscale values were quantitatively analyzed.

Additionally, the effects of lotus seed, jujube, and longan aril on the expression levels of NHE8 and AQP3 in colonic tissues from mice with senna leaf-induced diarrhea were further explored. Western blot results, depicted in Figure 3, indicated that, except for the Model + Longan Aril group, colonic AQP3 expression levels in the remaining treatment groups were higher than those in the Normal group. However, colonic NHE8 expression in the Model + Jujube and Model + Longan Aril groups was significantly lower than in the Normal group. These findings indicate that lotus seed, jujube, and longan aril have potential in repairing intestinal barrier damage in senna leaf-induced diarrhea (Table 10).

**Table 10:** Newly appeared gut bacteria after longan aril treatment compared to the union of control and model groups.

Species	Effects/Comments
*Altererythrobacter*	Involved in organic matter decomposition and nutrient cycling, contributes to carbon and nitrogen cycles (Ortega et al., 2024).
*Atopostipes*	/
*Bradyrhizobium*	/
*Deinococcus*	*Deinococcus radiodurans*, a radiation-resistant bacterium, exhibits antioxidant properties; its derived extracellular vesicles can act as potential radioprotectants by scavenging ROS, protecting bone marrow/spleen cells from radiation-induced death; protects intestinal stem/epithelial cells from radiation-induced apoptosis; stimulates SCFA production in GI tract, inhibits pro-inflammatory cytokines, increases Treg cells in pretreated mice; proteomics shows proteins involved in oxidative stress response are enriched (Han et al., 2025).
*Ensifer*	/
*Haloplasma*	/
*Herbaspirillum*	/
*Jeotgalibacillus*	/
*Marinilactibacillus*	/
*Paenisporosarcina*	Genomic content of this bacterium significantly reduced in rheumatoid arthritis patients (Ormseth et al., 2020).
*Proteus*	/
*Pseudomonas*	*Pseudomonas koreensis* promotes rice growth and reduces cadmium (Cd) content; Cd is easily absorbed by rice entering the food chain, posing human health risks (Wu et al., 2025).
*Reyranella*	Plays a key role in solid-phase denitrification; may be associated with high expression of ABC transporters and cytochrome P450 related to multidrug resistance and drug metabolism (Xie et al., 2022).
*Savagea*	Black soldier fly (BSF) larvae can convert organic waste and reduce zoonotic pathogens in the environment via gut-associated microbes like Savagea, e.g., inhibiting *Staphylococcus aureus* and Salmonella (Zhang et al., 2022).
*Shinella*	/
*Steroidobacter*	/

The discrepancy between AQP3 mRNA and protein expression in the jujube group may be attributed to post-transcriptional or post-translational regulatory mechanisms. Jujube’s unique active components, such as cAMP and specific polysaccharides, could influence AQP3 expression across multiple levels. Specifically, these components may promote AQP3 transcription by activating the PI3K/Akt signaling pathway, while simultaneously facilitating AQP3 protein degradation or inhibiting its translation, culminating in a net decrease in protein levels. This complex regulatory pattern highlights the substance-specific effects of medicinal and edible homology substances on water-electrolyte transporters.

### 3.5 16S rRNA analysis results of lotus seed on gut microbiota in senna leaf-induced diarrhea model mice

**Figure 4:**
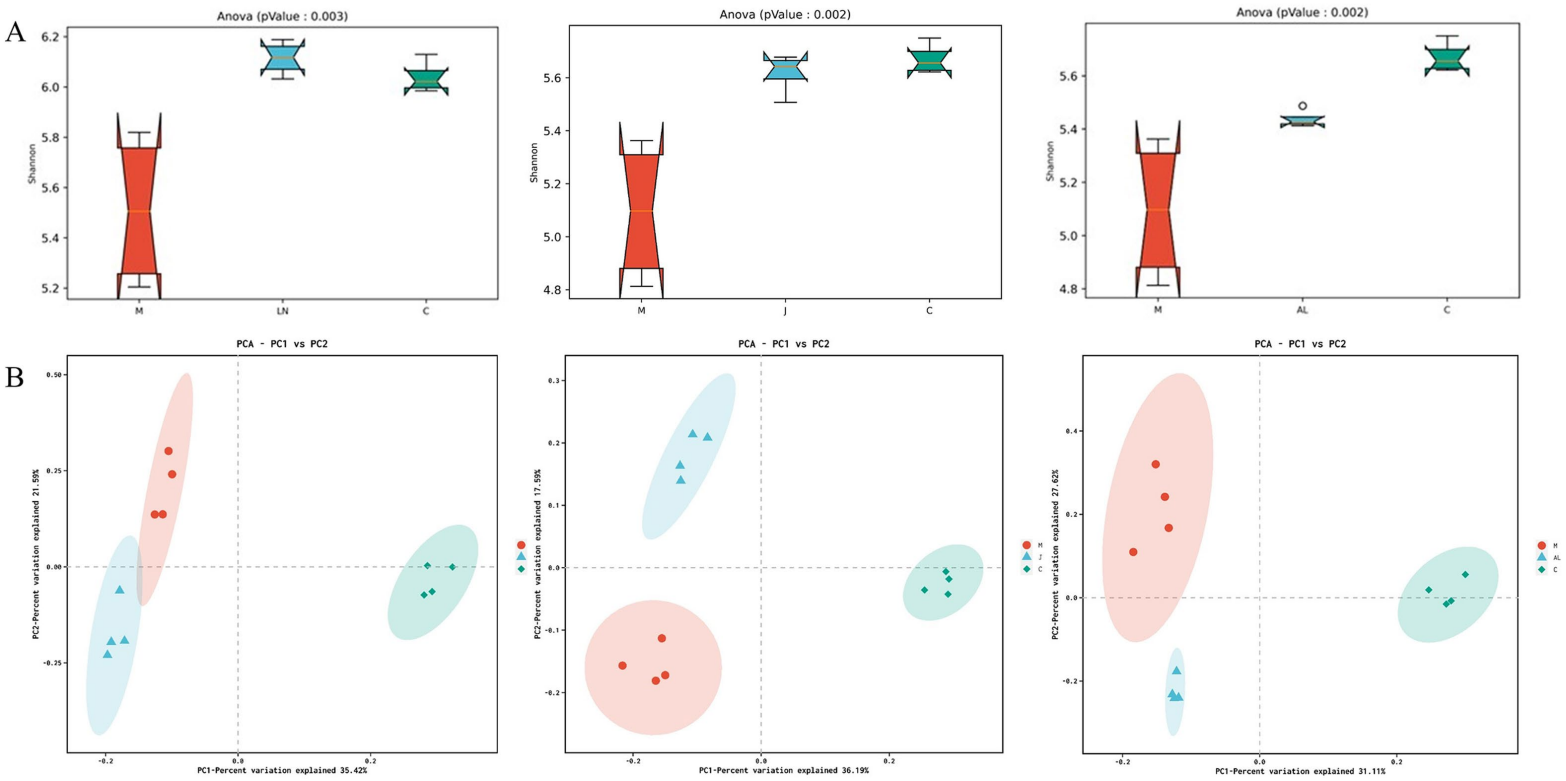
Analysis of gut microbiota in diarrheal mice (*α*-diversity and *β*-diversity). **(A)** Box plots of Shannon index demonstrating *α*-diversity of gut microbiota across experimental groups; **(B)** Principal component analysis (PCA) scatter plots illustrating *β*-diversity of gut microbial community structure across groups.

As illustrated in Figure 4A, groups C (healthy control), M (senna-induced diarrhea model), and LN (lotus seed), J (red jujube), or AL (longan aril) were consistently represented across box plots, PCA, stacked bar charts, and Venn diagrams. All three interventions significantly reduced gut microbial *α*-diversity (both richness and evenness associated with diarrhea), while treatment with lotus seed, red jujube, or longan aril effectively reversed this decline, restoring diversity to healthy levels. These findings provide microbiota-level evidence that each intervention reestablishes intestinal microecological stability by reinstating functional redundancy—the complementary metabolic and barrier-protective roles of various taxa. Consequently, the traditional efficacy of these foods in “relieving diarrhea and protecting the intestine” is supported by their ability to counteract dysbiosis and enhance gut physiological function.

Figure 4B presents PCA results of fecal gut microbiota, revealing distinct clustering patterns. In the lotus seed (LN) and red jujube (J) interventions, sample points from the model (M) and treatment groups clustered closely together, remaining distant from the normal control (C). This observation suggests that although *α*-diversity increased, the overall microbial structure still resembled that of the diarrheal model, indicating a “phased” regulatory effect—prioritizing the restoration of richness and evenness over complete architectural recovery. In contrast, the longan aril (AL) intervention demonstrated separate clustering among control, model, and treatment groups (PC1 and PC2 contributions: 31.11 and 27.62%, cumulative 58.73%). This indicates that longan aril not only restored α-diversity but also significantly reshaped the global microbial configuration, creating a composition distinct from both the normal and model groups. Thus, unlike lotus seed and jujube, longan aril achieves more comprehensive modulation—rebuilding both diversity and overall structure—highlighting its broader regulatory potential and providing essential insights into food-material specificity in microbiota modulation.

ANOVA bar charts at the species level (Supplementary Figure S1) revealed that treatment with lotus seed, red jujube, or longan aril selectively modulated the gut microbiota in diarrheal mice—upregulating some taxa while downregulating others, rather than inducing broad-spectrum changes. This targeted regulation suggests that these food materials may function by enhancing beneficial bacteria (e.g., short-chain fatty acid producers or barrier enhancers) while reducing diarrhea-associated or inflammatory pathogens. These findings confirm that as natural food-derived interventions, lotus seed, red jujube, and longan aril precisely correct pathological microbiota abnormalities without causing non-selective disruptions to gut microecology, highlighting their safety, specificity, and targeted advantages.

Venn diagram analyses at the genus level (Supplementary Figures S1, S2) indicated that lotus seed, red jujube, and longan aril each retained a significant proportion of core bacterial genera common to all three groups (137, 155, and 149, respectively). This finding confirms that neither diarrhea induction nor the interventions disrupted the essential gut microecological framework, thereby ensuring safety. For lotus seed, unique genera were 29 (Control), 19 (Model), and 15 (Lotus Seed); pairwise shared genera were 25 (C-M), 8 (C-LN), and 10 (M-LN). In the case of red jujube, unique genera amounted to 30 (C), 15 (M), and 9 (J), with pairwise shared genera totaling 7 (C-M), 7 (C-J), and 14 (M-J). For longan aril, unique genera included 26 (C), 18 (M), and 16 (AL), while pairwise shared genera were 13 (C-M), 11 (C-AL), and 11 (M-AL). All three interventions significantly reversed the diarrhea-induced decline in *α*-diversity (evidenced by an increased Shannon index) and selectively modulated specific taxa—upregulating short-chain fatty acid (SCFA) producers such as *Anaerotruncus colihominis* and *Blautia coccoides* for lotus seed, Faecalibaculum rodentium for jujube and longan aril, as well as Alloprevotella rava for longan aril, while downregulating pathogens including *Akkermansia muciniphila* across all treatments, plus Eisenbergiella tayi for longan aril. However, *β*-diversity analysis (PCA) revealed that the lotus seed and jujube groups remained clustered with the model group, indicating “partial restoration,” where *α*-diversity improved but the overall structure was not fully reverted. Conversely, the longan aril group formed a distinct cluster apart from both control and model groups, signifying “comprehensive reshaping” of the microbial architecture. Notably, lotus seed eliminated Citrobacter and introduced Azospirillum; jujube eliminated Anaerofilum and introduced Lachnoanaerobaculum; longan aril removed Anaerofilum, gained 16 unique genera (including Lachnoanaerobaculum), and introduced the antioxidant Deinococcus. Collectively, senna-leaf-induced diarrhea has a tripartite pathological impact characterized by reduced α-diversity, structural shift, and compositional abnormalities. Lotus seed and red jujube promote adaptive, phased optimization without disrupting core taxa, while longan aril achieves both diversity restoration and overall structural remodeling, resulting in a microbiota composition specifically tailored for alleviating diarrhea. These findings support the development of these natural food materials as safe and targeted modulators of intestinal microecology.

## 4 Discussion

This study systematically explored the anti-diarrheal effects of three medicinal and edible substances—lotus seed (*Nelumbo nucifera*), jujube (*Ziziphus jujuba*), and longan aril (*Dimocarpus longan*)—utilizing a senna leaf-induced diarrhea mouse model. Phenotypically, all three substances significantly reduced the incidence of loose stools and diarrhea index, while also inhibiting small intestinal hypermotility, indicating effective regulation of intestinal motility. Concurrent histopathological and immunohistochemical analyses demonstrated restoration of intestinal barrier integrity, marked by increased goblet cell proliferation and enhanced expression of tight junction proteins. These collective findings suggest a multi-dimensional anti-diarrheal effect that encompasses both motility regulation and barrier repair.

However, this study employed only a single dosage for each intervention, lacking a dose gradient design to thoroughly investigate the dose–response relationship. Although the selected dose exhibited significant therapeutic effects in preliminary experiments and aligned with previous research, future investigations should incorporate multiple dosage groups to establish the optimal therapeutic dose and elucidate the dose-dependent effects of these substances on gut microbiota and intestinal barrier function.

16S rRNA gene sequencing revealed that all three substances effectively remodeled the gut microbiota of diarrheal mice, albeit with distinct patterns. Regarding *α*-diversity, lotus seed, jujube, and longan aril all significantly reversed the diarrhea-induced decrease in the Shannon index (approximately 20% increase, *p* < 0.05). In terms of *β*-diversity, longan aril exhibited a more comprehensive impact on overall microbial community structure, while lotus seed and jujube displayed selective changes primarily in α-diversity and specific taxa. Notably, the relative abundance of the potentially pathogenic *Akkermansia muciniphila*, associated with mucosal damage, and the opportunistic pathogen Citrobacter significantly decreased with all three substances (*p* < 0.01). The lotus seed group remarkably reduced Citrobacter levels to below the detection limit of 16S sequencing, whereas the montmorillonite control group achieved only a roughly 50% reduction in relative abundance. In contrast, all three substances enriched beneficial taxa, illuminating a directionally increased presence of short-chain fatty acid (SCFA)-producing bacteria, including *Blautia coccoides* and Faecalibaculum rodentium (*p* < 0.05). Additionally, unique taxa emerged, such as nitrogen-fixing Azospirillum in the lotus seed group and antioxidant Deinococcus in the longan aril group, highlighting substance-specific functional synergies. At the molecular level, all three substances significantly reduced AQP3 protein expression in colonic tissues, though jujube exhibited a divergent effect on AQP3 mRNA expression (*p* < 0.05). Additionally, lotus seed and longan aril further upregulated sodium–hydrogen exchanger 8 (NHE8) (Xu et al., 2015). This dual regulation of water-electrolyte transport was associated with the restoration of tight junction proteins (ZO-1, occludin). A cohesive model is proposed wherein SCFAs derived from enriched beneficial bacteria (e.g., Blautia and Faecalibaculum) act as key mediators. SCFAs are known to activate GPR41/43 receptors, which can suppress AQP3 overexpression and stimulate NHE8-mediated sodium absorption while concurrently enhancing tight junction integrity and diminishing pro-inflammatory cytokines. Thus, the “microbiota-SCFA-barrier” axis integrates microbiome shifts with molecular events, establishing a functional cascade from microbial remodeling to phenotypic improvement.

Based on their distinct regulatory profiles, each substance may prove beneficial in the clinical management of diarrhea: Lotus seed exhibited the most potent effect against Citrobacter, with relative abundance falling below the detection limit. This characteristic suggests its potential advantage in cases where opportunistic pathogens are significant contributors to diarrhea or in contexts involving drug-resistant bacterial strains. Jujube, while also effective in reducing pathogenic taxa, demonstrated a more pronounced trend toward the upregulation of NHE8, indicating potential benefits for diarrhea subtypes characterized by significant electrolyte loss, where enhanced sodium absorption is clinically desirable. Longan aril showcased the most extensive remodeling of *β*-diversity and uniquely enriched antioxidant bacteria such as Deinococcus; thus, it may be particularly suitable for diarrheal conditions associated with oxidative stress or severe microbial dysbiosis.

In contrast to traditional antidiarrheals (e.g., montmorillonite) or probiotic preparations with limited colonization rates, these medicinal and edible substances function as natural prebiotics (e.g., polysaccharides) that promote the proliferation of endogenous beneficial microbiota, offering a more sustainable and multi-target regulatory approach.

Despite the integrated multi-dimensional methodology, this study has certain limitations. Firstly, the mechanistic depth is insufficient; the proposed regulatory pathways involving AQP3 and NHE8 have not been verified using gene knockout models, and the causal relationships between specific microbial taxa and molecular changes require further validation. Secondly, metabolomic analyses of SCFAs, bile acids, and other key metabolites were not conducted, limiting demonstration of the microbiota-host metabolic crosstalk. Future studies should integrate genetic and metabolomic approaches to enhance mechanistic conclusions and optimize dosing regimens for clinical applications.

## 5 Conclusion

In conclusion, this study illustrates that lotus seed, jujube, and longan aril alleviate diarrhea through a multi-target “microbiota-intestinal barrier” regulatory axis, with each substance exhibiting distinct modulation patterns and potential subtype-specific clinical applications. These findings provide an experimental foundation for the precise use of medicinal and edible substances in diarrhea management.

## Data Availability

The datasets presented in this study can be found in online repositories. The names of the repository/repositories and accession number(s) can be found in the article/Supplementary material.

## Supplementary material

SUPPLEMENTARY FIGURE S1 Analysis of gut microbiota in diarrheal mice (species difference analysis and marker species).

SUPPLEMENTARY FIGURE S2 Analysis of gut microbiota in diarrheal mice (species composition analysis).

SUPPLEMENTARY FIGURE S3 Time-dependent effects of lotus seed, jujube, and longan aril at different doses on the diarrhea index in mice.

SUPPLEMENTARY FIGURE S4 Time-dependent effects of Berberine hydrochloride at different doses on the diarrhea index in mice.
