# Supplementary figures and images for: Modulation of gut microbiota and intestinal barrier by lotus seed, jujube, and longan aril in senna leaf-induced diarrhea in mice
Source: Front Microbiol. 2026 Jul 7;17:1796355. doi: 10.3389/fmicb.2026.1796355 (PMC13385037; doi:10.3389/fmicb.2026.1796355)

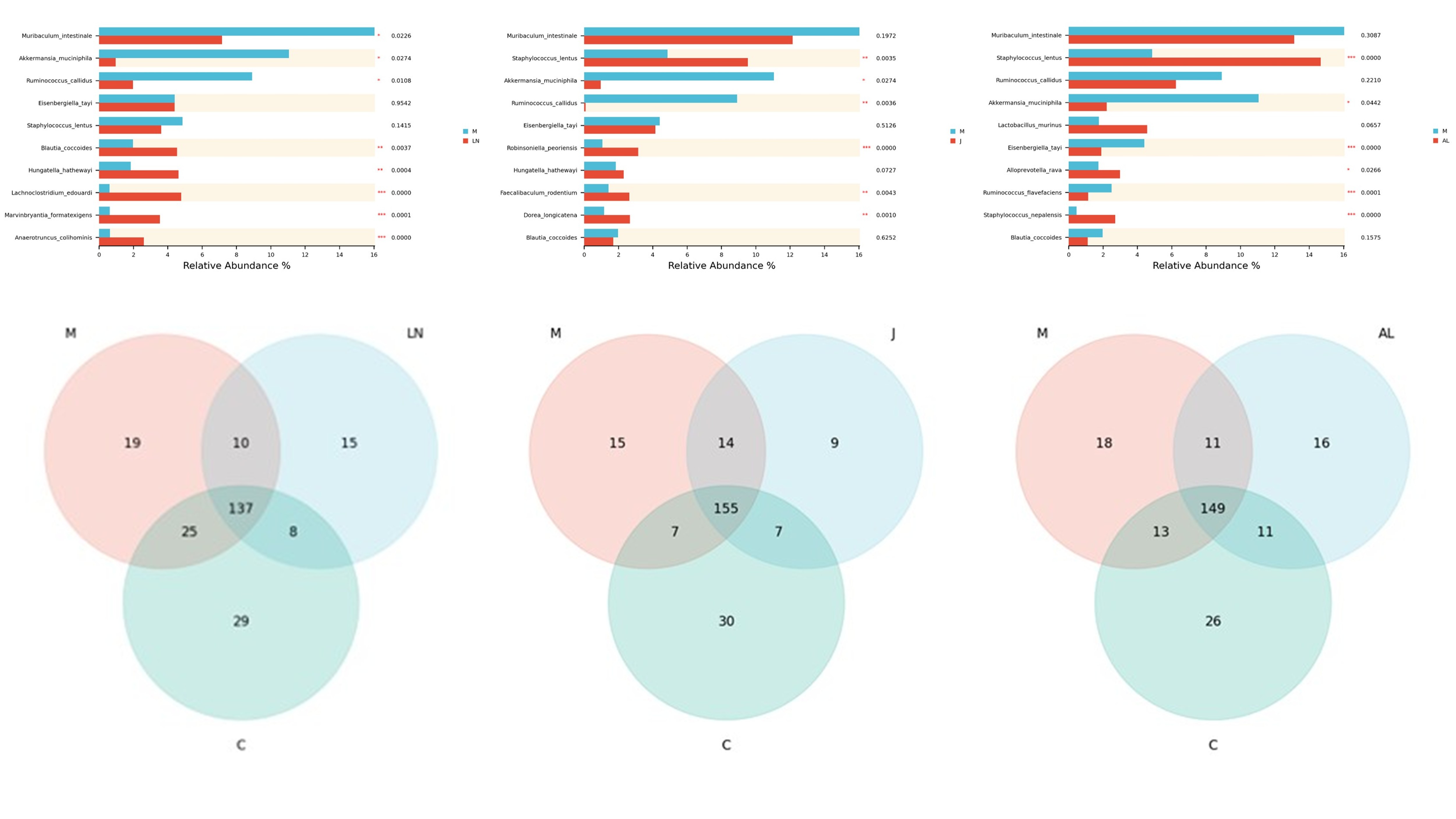

Supplement: SUPPLEMENTARY FIGURE S1 — Analysis of gut microbiota in diarrheal mice (species difference analysis and marker species). [file Image_1.JPEG]

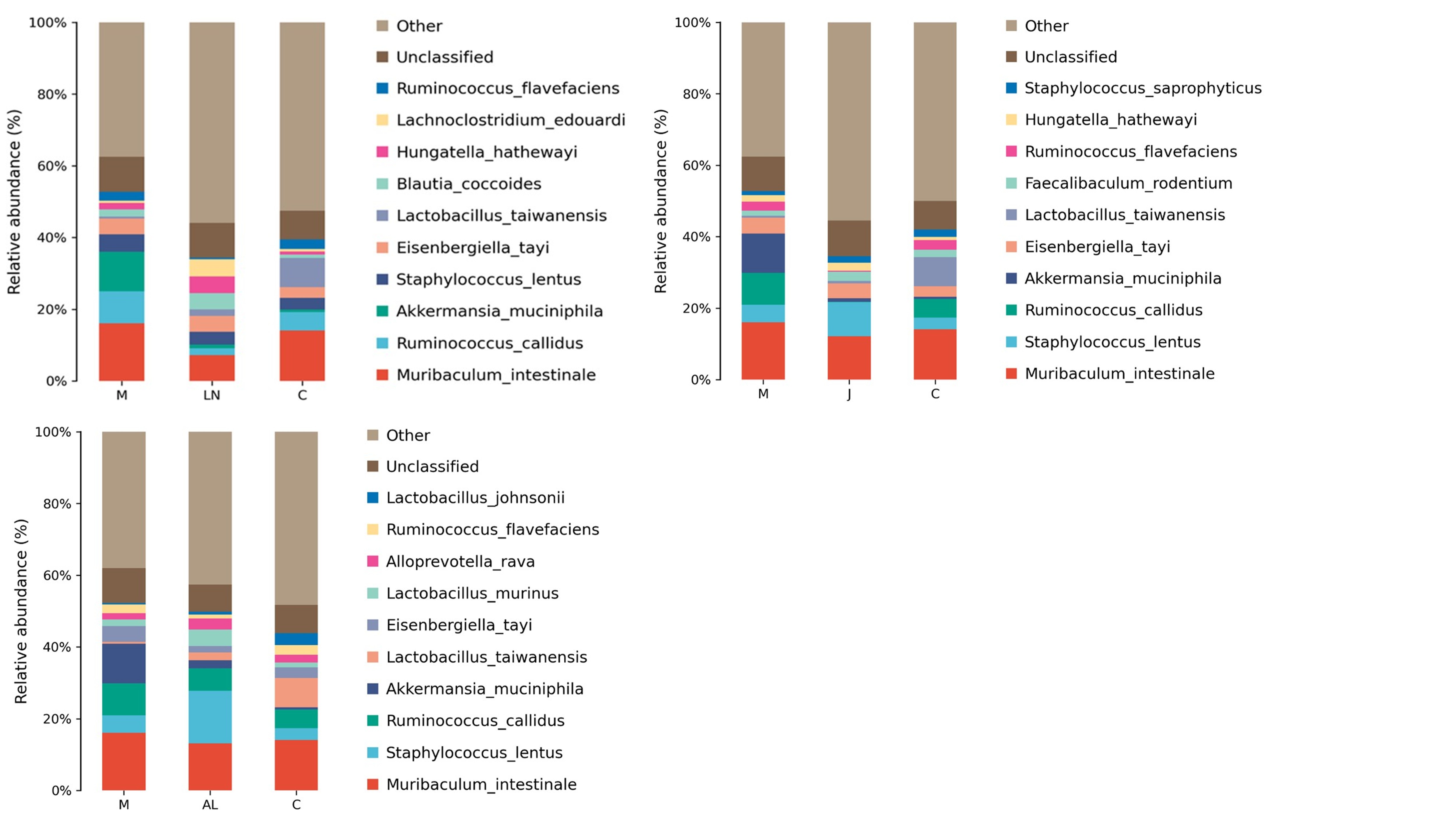

Supplement: SUPPLEMENTARY FIGURE S2 — Analysis of gut microbiota in diarrheal mice (species composition analysis). [file Image_2.JPEG]

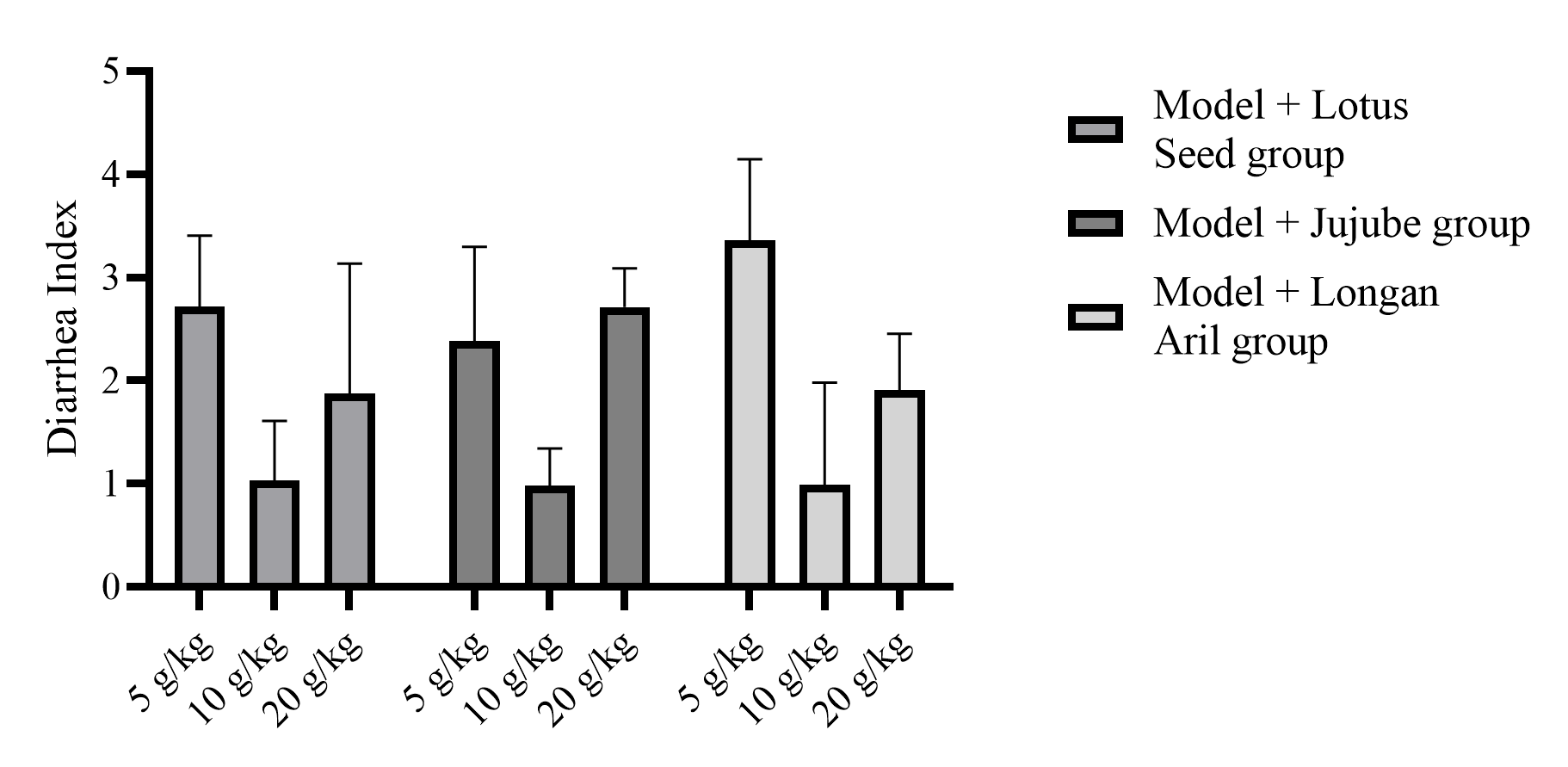

Supplement: SUPPLEMENTARY FIGURE S3 — Time-dependent effects of lotus seed, jujube, and longan aril at different doses on the diarrhea index in mice. [file Image_3.TIF]

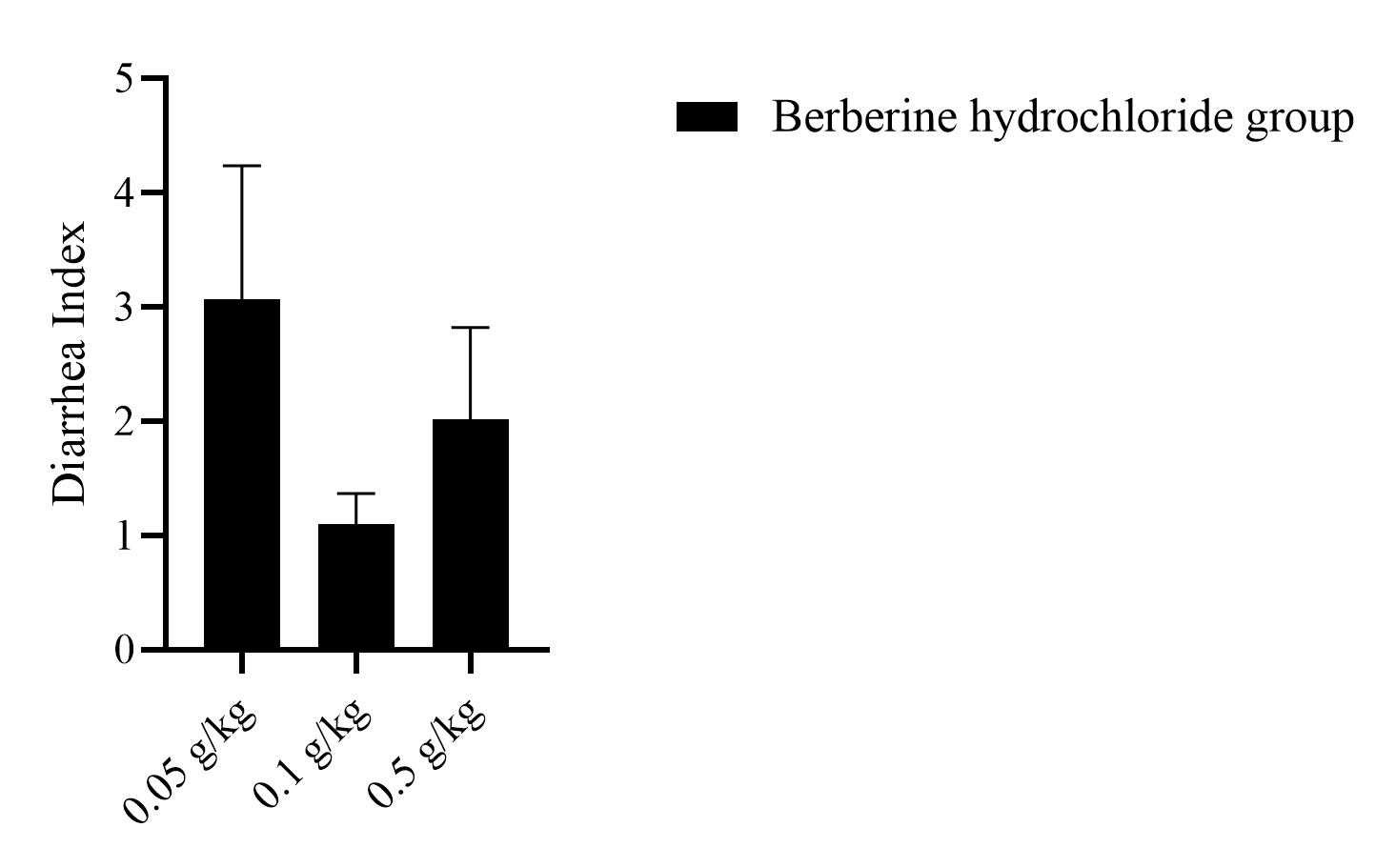

Supplement: SUPPLEMENTARY FIGURE S4 — Time-dependent effects of Berberine hydrochloride at different doses on the diarrhea index in mice. [file Image_4.TIF]
